# Bi-objective inventory allocation planning problem with supplier selection and carbon trading under uncertainty

**DOI:** 10.1371/journal.pone.0206282

**Published:** 2018-11-28

**Authors:** Kai Kang, Wei Pu, Yanfang Ma, Xiaoyu Wang

**Affiliations:** School of Economics and Management, Hebei University of Technology, Tianjin, P. R. China; Southwest University, CHINA

## Abstract

Concern is growing that business enterprises focus primarily on their economic activities while disregarding the adverse environmental and social effects of these activities. To contribute to the literature on this matter, this study investigates a novel bi-objective inventory allocation planning problem with supplier selection and carbon trading across multiple periods under uncertainty. The concepts of a carbon credit price and a carbon cap are proposed to demonstrate the effect of carbon emissions costs on inventory allocation network costs. Demands of manufacturers, transport price, and defect rate of materials that should be rejected are set as random variables. We combine normalized normal constraint method, differential evolution algorithm, and uncertainty simulation to deal with the complex model. One representative case shows the effectiveness and practicability of this model and proposed method. The Pareto frontier is generated by solving the bi-objective model. We extend the results of numerical examples in large scale problems, and compare the solution method results with exact solutions. The environmental objective across the inventory allocation network varies with changes of the carbon cap and the carbon credit price.

## Introduction

The need for environmental awareness has influenced the worldwide economy, including supply chain network planning. Owing to the urgency of global warming, low-carbon economic activities have attracted widespread attention. How supply chain managers make decisions to balance operating costs and low-carbon efficiency under carbon cap and carbon trading mechanism has become an important research field that highlights the need to achieve carbon emission reduction. As the global low carbon emission reduction becomes an increasingly serious matter, the uncertainty and complexity of the environment make higher demands on the flexibility and efficiency of the supply chain management. Inventory allocation management considering low carbon under uncertainty has become a proposition that needs to be studied in the process of value creation in supply chain.

In the logistics project, the transportation process is the main process of producing carbon emissions. Optimal decision making through model optimization is the common plan of supply chain. Several models has been established and studied previously [1–4]. Moncer and Rami [5] presented a carbon footprint minimization model, an operational cost minimization model, and a hybrid economic and environmental minimization model to determine the optimal lot sizing and allocation quantities. Tien and Bhaba [6] designed a pull system inventory with imperfect-quality items; their study investigates various tax systems and numerical cases illustrate that the different carbon tax policies lead to different inventory strategies, and the lot size and order frequency depend on the discount policies and the carbon tax systems. Zhang and He [7] proposed a multi-modal logistics model with time window to minimize total cost and carbon emissions cost by determining the optimal transportation mode and investment selections. Demir and Tolga [8] studied the bi-objective pollution vehicle routing problem, and proposed an effective trade-off between the two contradictory objective of minimizing fuel consumption and delivery time. Li and Su [9] investigated three carbon policies: carbon tax policy, cap-and-trade policy, and joint cap-and-trade policy. They constructed models under different carbon policies and optimal solutions were obtained. Kyoto Protocol, a worldwide agreement ratified by the United Nations, is the most prominent international framework that outlines emissions trading schemes and a carbon credit market to minimize carbon emissions [10].

**Fig 1:**
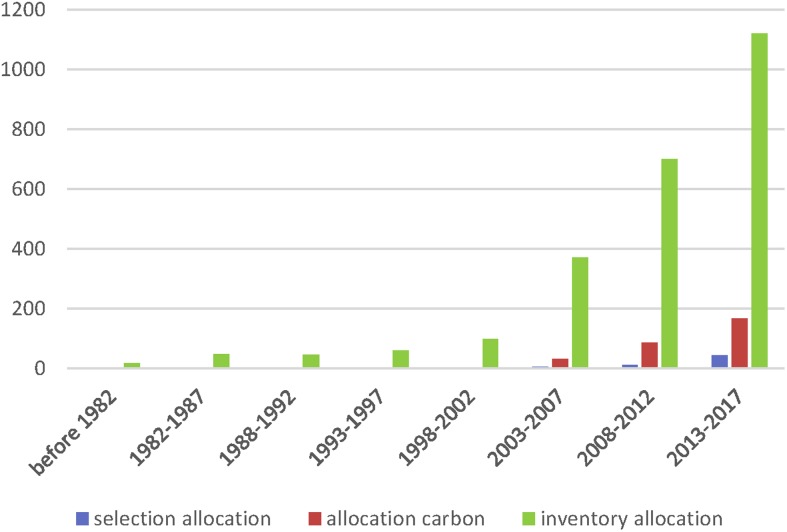
The comparison of the annual distribution of key words.

An uncertain competitive environment requires inventory allocation management to be flexible and efficient. Enterprises need to not only reduce their storage and distribution cost, but also ensure that downstream manufacturers are not unduly affected by out-of-stock materials at a critical time. Without proper control, interests will be damaged and the entire supply chain may disrupt [11]; therefore, supplier selection, supply chain inventory and distribution management have become important to supply chain efficiency [12–14]. Armin and Saba [15] proposed a bi-objective mathematical model to maximize the score of all sustainable suppliers according to preference weights and minimize the total cost. Kijung et al. [16] applied an information-based multi-attribute and multi-objective decision-making approach for supplier selection and order allocation. Seda and Ender [17] proposed a multi-objective optimization model for supplier selection and inventory planning, which minimizes the conflicting objectives of operation cost and supplier risk. Time management has also become crucial, especially when multiple time periods are involved [18]. Rezaei et al. [19] extended the multi-objective non-linear mixed integer models for multi-period allocation planning problems that involved multiple suppliers and materials which may be defective obey the probability distribution. Khan et al. [20] proposed an inventory model with single supplier and single manufacturer to evaluate the effect of varying percentages of defective goods, storage costs, and disposal schemes. To better analyze the current research, we used NoteExpess to analyze the literatures in Web of Science with the key words: selection allocation, low carbon allocation, inventory allocation. The comparison of the annual distribution of these key words were presented in Fig 1. It can be seen from the Fig 1 that the inventory allocation planning problem considering carbon emissions and supplier selection is getting more and more attention.

In the traditional inventory allocation model, important parameters, such as manufacturers’ demand, transport price, and defect rate are set as certain values. However, the weather, traffic, and equipment failures are typical uncertainties, which are considered to be random. When these parameters have the random nature with a known distribution, the randomness uncertainty is used. The stochastic programming is the most common method to face this uncertainty [21, 22]. When historical data is available and we can estimate probability distribution of uncertain parameters, stochastic programming is used as the mathematical programming. Uncertainty mentioned above exist in the inventory allocation problem. Thus, the manufacturers’ demand, transport price and defect rate are set as random variables.

In the current study, a bi-objective inventory allocation planning model with supplier selection and carbon trading (IAPSSCT) under uncertainty was proposed. Random variables included demands of manufacturers, transportation price, and material defect rate. In bi-objective optimization, one solution cannot satisfy every objective, simultaneously. Previous research provides a variety of methods to generate the Pareto frontier [23], such as weighting method, *ϵ*-constraint method, and normalized normal constraint method. Normalized normal constraint (NNC) method, proposed by Messac et al. [24], obtains an evenly spaced Pareto solution by constructing a series of single-objective optimization problems after normalized processing of the feasible region of multi-objective optimization [25]. Among numerous heuristic and metaheuristic algorithms, the differential evolution (DE) algorithm has been proven to be a competitive contender for NP-hard optimization, such as logistics management [26] and production scheduling [27], as well as in the field of multi-level and multi-period optimization [28, 29]. The selection of fundamental control parameters including population size *L*, crossover probability *CR*, and mutation scale factor *F*, will severely affect the convergence of basic DE. In this condition, the basic DE can easily to fall into local minima, and convergence slows down. The core procedure to improve DE performance is to select the appropriate mutation strategy and control parameters. The mutation operator is then improved to make the results effectively drop out of local regions and converge rapidly to the optimal direction [30].

The followings are the main contributions of this study. (1) A bi-objective inventory allocation planning model with supplier selection and carbon trading (IAPSSCT) under uncertainty is proposed to find the trade-off between economic and environmental objective by determining supplier selection, purchase quantity, inventory quantity, and allocation quantity. (2) Given the subjective and objective uncertainties in reality, the manufacturers’ demand, transport price, and defect rate are considered as random variables, which characterizes the uncertainty of the inventory allocation problem. (3) To address the complexity of the model, Pareto frontier is generated by NNC–DE. (4) By analyzing the simulation results, we discussed the impact of the carbon cap and carbon credit prices on environmental objective and inventory allocation decisions, and provide suggestions for decision makers.

The rest of this paper is organized as follows: Section 2 presents the key problem statement and problem assumptions. Section 3 proposes the mathematical bi-objective IAPSSCT model under uncertainty. The algorithm combined with NNC and DE is proposed in Section 4. A case study is presented in Section 5 to verify the effectiveness and efficiency of the model, and the influence of carbon cap and carbon credit price on the environmental objective and the decisions of inventory allocation are discussed. Finally, Section 6 is the conclusion of this paper and the directions for future research.

## Key problem statement

Inventory allocation management with good quality and carbon emission control is essential in a supply chain to achieve an efficient supplier–manufacturer network. This inventory allocation problem involves multiple suppliers that can provide multiple materials. Different suppliers offer the same types of materials with defect rates at varying prices. One type of material should be purchased from one supplier, and one supply hub stores one type of materials; thus, the number of supply hubs is the same as the number of materials types. In this system, multiple manufacturers order all types of materials.

**Fig 2:**
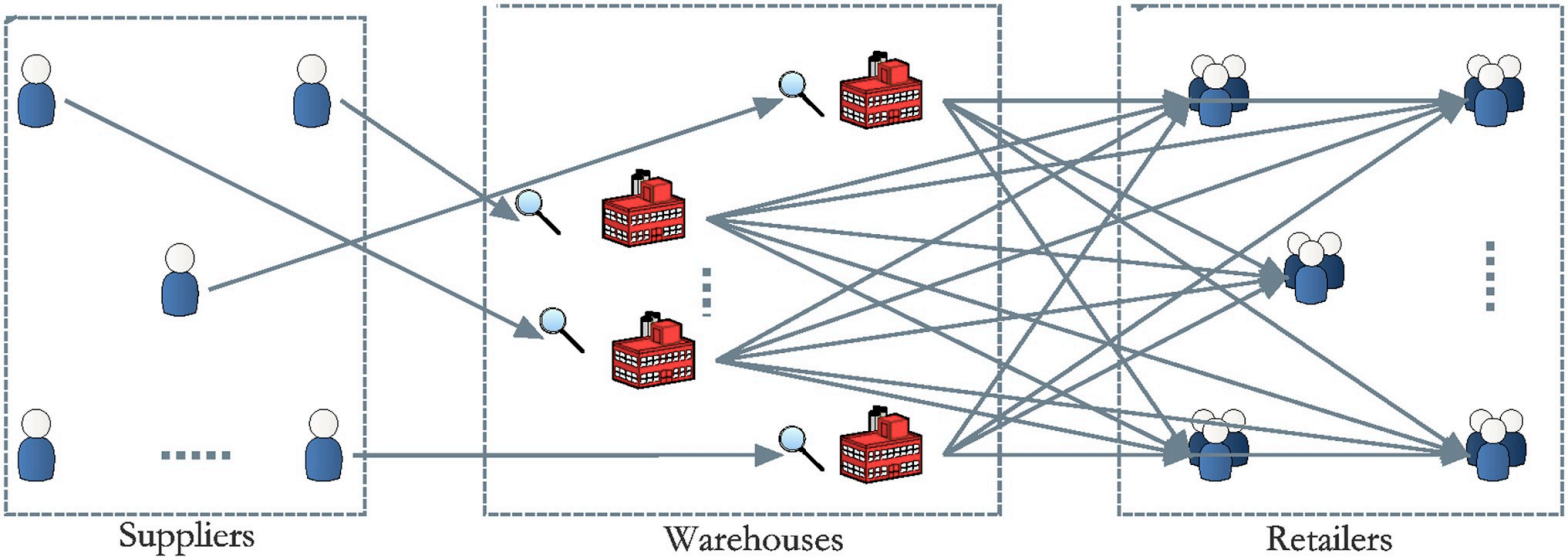
The flow of materials in supplier–manufacturer network.

In many inventory allocation models, all materials are deemed to be of suitable quality; in the real world, however, several materials are defective, and the percentage is uncertain. In this supplier–manufacturer network, purchase, inventory, and allocation are periodical, each period includes purchase, inventory, and allocation process [31, 32]. The flow of materials is described in Fig 2. At the beginning of the period, supply hub operator select proper suppliers where they purchase materials. Suppliers deliver the materials to the corresponding supply hub, and the materials are placed in stores after being inspected. The inspection entails classifying materials as suitable or defective. The defective materials found during the screening process are returned to the suppliers. For convenience, the suppliers take back the defective materials as a batch in the next period [33, 34]. When defective materials are present, materials shortage is likely to occur. Therefore, a penalty cost is considered for the loss due to the possible shortage. All materials are arranged and then distributed to each manufacturer according to their demand.

In the supplier–manufacturer network considered in this study, the manufacturers’ demands may be affected by weather, emergencies, and other factors, and may also be uncertain because of the manufacturers’ subjective judgment. In actual logistic transportation, weather, traffic congestion, and unexpected events are the main objective factors that affect the transportation cost and these factors are considered random. In addition, the defect rate is uncertain due to the complexity of the production process and instability of the material transportation and loading process. Thus, the manufacturers’ demands, unit transport price and defect rate are set as random variables.

The main factor for carbon emissions is transport emission, and the carbon emissions which generated from suppliers’ plants usually reflect in the pricing of materials. The price of materials includes cost of carbon emissions in raw materials processing [35–37]. Thus, the transportation-related emissions have been focused in this inventory-allocation problem. The transportation distance is a direct influence factor of environmental objective, and the location of suppliers directly affects the transportation distance of suppliers to the supply hub. Consequently, the suppliers’ selection is the indirected factor that affects the environmental objective.

With environmental regulations on carbon cap by the government, carbon emissions from transportation should be kept below a certain level. Through the carbon cap and carbon trading, carbon emissions can be measured by economic indicators. The environmental objective of the proposed supplier–manufacturer network depends on whether the allocation of carbon footprint is in accordance with the established carbon emissions reduction targets. At the end of the final period, the actual carbon emission is measured, and the emissions that exceeded the appointed target are offset by the carbon footprint that the company purchased from carbon trading market [38]. For the remaining carbon emissions, alternatively, the manufacturer earns carbon credits that can be sold in the carbon market. The excess carbon emissions emitted in the process of transportation become the cost of the supplier–manufacturer network, and the remainder is converted into the supplier–manufacturer network revenue.

The following assumptions are made in this study: (1) Material demands, transportation costs, and the defect rate of material in each period are set as random variables [18]. (2) The time span of each period is indistinguishable [17]. (3) Shortage is allowed and a penalty cost is applied to diminish economic losses on account of shortages [39]. (4) Suppliers who have been selected are responsible for the costs incurred in returning defective materials [35]. (5) Every material type has a corresponding supply hub with a maximum storage capacity [40]. (6) The order lead time is negligible [37]. (7) Manufacturers’ material demands are independent of one another and are fixed within a period [18].

## Modeling

A mathematical model formulation for IAPSSCT that considers randomness is constructed in this section.

### Notations

To facilitate our model, notations are defined as follows:

**Indices**
*i*: index for periods; *i* ∈ Ψ = {0, 1, …, *I* − 1}
*t*: index for materials; *t* ∈ Φ = {1, 2, …, *T*}
*s*: index for suppliers; *s* ∈ Θ = {1, 2, …, *S*}
*n*: index for manufacturers; *n* ∈ Ω = {1, 2, …, *N*}
*m*: price break point index; *m* ∈ Π = {1, 2, …, *M*}

**Decision variables**
*w*_*tis*_: a binary variable indicating whether a supplier is chosen. If supplier *s* is chosen, then *w*_*tis*_ = 1; otherwise, *w*_*tis*_ = 0
*x*_*tis*_: purchase quantity of material *t* from supplier *s* in the (*i* + 1)^*th*^ period
*l*_*ti*_: stock level of material *t* in the beginning of the (*i* + 1)^*th*^ period
*y*_*tin*_: allocation quantity of material *t* from supply hub *t* to manufacturer *n* in the (*i* + 1)^*th*^ period

**Parameters**
$\bar{\gamma_{ti}}$: unit transport price of material *t* per kilometer in the (*i* + 1)^*th*^ period
$\bar{\mu_{tin}}$: demand for material *t* at manufacturer *n* in the (*i* + 1)^*th*^ period
$\bar{u_{ti}}$: demand for material *t* in the (*i* + 1)^*th*^ period
$\bar{q_{ts}}$: defect rate of material *t* from supplier *n* in the (*i* + 1)^*th*^ period
$P_{t}^{max}$: maximum purchase quantity of material *t* in the (*i* + 1)^*th*^ period
$P_{t}^{min}$: minimum purchase quantity of material *t* in the (*i* + 1)^*th*^ period
$Q_{t}^{max}$: maximum stocks limit of material *t*
*S*_*t*_: original stock level of material *t* at the beginning of the initial period
*T*_*t*_: final stock level of material *t* at the end of the entire process
*K*_*t*_: unit holding cost of material *t*
*L*_*t*_(*s*): actual stock function for material *t* in the entire process
*D*_*t*_: distance between supplier *t* and the corresponding supply hub
*D*_*tn*_: distance between supply hub *t* and manufacturer *n*
*d*_*t*_: inspection fee of material *t*
*r*_*t*_: unit refund of defective material *t*
*σ*_*t*_: unit shortage fee of defective material *t*.
*c*_*tm*_: unit cost of material *t* from a supplier at *m*^*th*^ price break point
*s*_*tm*_: *m*^*th*^ price break point for the material *t* in the (*i* + 1)^*th*^ period
*B*: total budget of a manufacturer for the planning horizon
*ξ*_*c*_: fuel consumption per kilometer for transportation vehicle
*ξ*_*e*_: *CO*_2_ emission of unit gasoline fuel for transportation vehicle
*C*: carbon cap over the network
*ϖ*: carbon credit price per ton

### Objective functions

The model consists of economic and environmental objectives.

#### Economic objective

The economic objective function describes the cost of the entire supplier–manufacturer network. The goal of a supply hub operator is to determine the supplier selection, purchase quantity, inventory level, and allocation quantity of each material in each period to minimize the total supplier–manufacturer network costs. The total network costs comprise of purchasing, inventory, penalty, and transportation costs.

In general, a manufacturer orders materials under several discount policies [41]. In this model, the incremental quantity discount policy (IQD) is considered. In the IQD, the purchase costs of material *t* in the corresponding period depend on the purchased amount. The price discount point can be described as:

$$\begin{array}{cc} & s.t.\{\begin{array}{c} c_{t1}\;\;\;\;\;\;\;\;\;\;\;\;\;\;\;\;\;\;\;\;\;\;\;\;\;\;\;s_{t1}\leq x_{tis}<s_{t2}\\ c_{t1}s_{t1}+\left(x_{tis}-s_{t2}\right)c_{t2}\;\;\;\;\;\;\;\;\;\;s_{t2}\leq x_{tis}<s_{t3}\\ \;\;\vdots \;\;\;\;\;\;\;\;\;\;\;\;\;\;\;\;\;\;\;\;\;\;\;\;\;\;\vdots \\ c_{t1}s_{t1}+c_{t2}s_{t2}+\cdots +c_{tM}\left(x_{tis}-s_{tM}\right)\;\;s_{tM}\leq x_{tis}. \end{array} \end{array}$$

If the supplier is chosen, then *w*_*tis*_ = 1. Therefore, the purchase cost (*F*^*PC*^) under IQD is

$$\begin{array}{c} F^{PC}=\sum_{s}\sum_{t}\sum_{i}\left[\sum_{m}\left(s_{tm+1}-s_{tm}\right)c_{tm}+\left(x_{tis}-s_{tM}\right)c_{tM}\right]w_{tis}\\ \forall s\in \Theta ,\;i\in \Psi ,\;t\in \Phi ,\;m\in \Pi . \end{array}$$ (1)

Materials with the defect rate, $\bar{q_{ts}}$, are inspected before being transported to the supply hub, after which all defective materials are returned to the supplier and a return cost is requested. Let *F*^*IC*^ be the inventory cost, ∑_*t*_∑_*i*_ *K*_*t*_*l*_*ti*_ be the holding cost, ∑_*s*_∑_*t*_∑_*i*_ *x*_*tis*_*w*_*tis*_ *d*_*t*_ be the inspection cost, and $\sum_{s}\sum_{t}\sum_{i}x_{tis}w_{tis}\bar{q_{ts}}r_{t}$ be the return cost when defective materials are returned to the corresponding suppliers. Thus,

$$\begin{array}{c} F^{IC}=\sum_{t}\sum_{i}K_{t}l_{ti}+\sum_{s}\sum_{t}\sum_{i}x_{tis}w_{tis}d_{t}-\sum_{s}\sum_{t}\sum_{i}x_{tis}w_{tis}\bar{q_{ts}}r_{t}\\ \forall i\in \Psi ,\;t\in \Phi . \end{array}$$ (2)

The transportation distances between suppliers, supply hubs, and manufacturers vary, so does supplier selection in different periods. $\sum_{s}\sum_{t}\sum_{i}\bar{\gamma_{ti}}D_{t}x_{tis}w_{tis}$ calculates the delivery cost from selected suppliers to supply hubs, and $\sum_{t}\sum_{i}\sum_{n}\bar{\gamma_{ti}}D_{tn}y_{tin}$ calculates the delivery cost from supply hubs to every manufacturer. If manufacturers’ demands can be satisfied, then $y_{tin}=\bar{\mu_{tin}}$; thus, transportation cost (*F*^*TC*^) is

$$\begin{array}{c} F^{TC}=\sum_{s}\sum_{t}\sum_{i}\bar{\gamma_{ti}}D_{t}x_{tis}w_{tis}+\sum_{t}\sum_{i}\sum_{n}\bar{\gamma_{ti}}D_{tn}y_{tin}\\ \forall s\in \Theta ,\;i\in \Psi ,\;t\in \Phi ,\;n\in \Omega . \end{array}$$ (3)

A penalty cost is applied when the demand for material *t* cannot be satisfied. $\bar{u_{ti}}-l_{ti}-\left(1-\bar{q_{ts}}\right)x_{tis}$ is the shortage quantity of material *t* in period *i*, and *σ*_*t*_ is the unit shortage cost. Let *F*^*PeC*^ be the penalty costs for material *t*, which can be determined as follows:

$$\begin{array}{c} F^{PeC}=\sum_{s}\sum_{t}\sum_{i}\sigma_{t}w_{tis}\left[\bar{u_{ti}}-l_{ti}-\left(1-\bar{q_{ts}}\right)x_{tis}\right]\;\forall s\in \Theta ,\;i\in \Psi ,\;t\in \Phi . \end{array}$$ (4)

The economic objective function of inventory allocation network is

$$\begin{array}{cc} & minF_{1}=min\left[F^{PC}+F^{IC}+F^{TC}+F^{PeC}\right], \end{array}$$ (5)

#### Environmental objective

The environmental objective is to minimize the carbon emission during the transportation process. The environmental objective under the carbon cap and trade policy are the penalties/rewards in carbon-constrained scenario. ∑_*s*_∑_*t*_∑_*i*_ *D*_*t*_*ξ*_*c*_*ξ*_*e*_*x*_*tis*_ *w*_*tis*_ represents transport emissions from suppliers to supply hubs, ∑_*s*_∑_*t*_∑_*i*_ *D*_*t*_*ξ*_*c*_*ξ*_*e*_*x*_*tis*_*q*_*ts*_ *w*_*tis*_ represents transport emissions for defective materials from supply hubs to corresponding suppliers, and $\sum_{t}\sum_{i}\sum_{n}D_{tn}\xi_{c}\xi_{e}\bar{\mu_{tin}}$ represents transport emissions from supply hubs to every manufacturer. As defined, *ξ*_*c*_ is the fuel consumption (*l*/*km*) of a transportation vehicle, and *ξ*_*e*_ is the *CO*_2_ emission (*kg*/*l*) from gasoline. Thus, the carbon emissions of vehicles per kilometer are denoted by *ξ*_*c*_*ξ*_*e*_(*kg*/*km*). *C* is the carbon cap during transport, and the carbon credit price (*ϖ*) should be considered for the purchase and sale of carbon credits [40, 42]. Therefore, *F*_2_ is the carbon emissions costs of material *t* across the entire supplier–manufacturer network as follows:

$$\begin{array}{c} minF_{2}=\varpi (\sum_{s}\sum_{t}\sum_{i}D_{t}\xi_{c}\xi_{e}x_{tis}w_{tis}+\sum_{s}\sum_{t}\sum_{i}D_{t}\xi_{c}\xi_{e}x_{tis}q_{ts}w_{tis}\\ \;+\sum_{t}\sum_{i}\sum_{n}D_{tn}\xi_{c}\xi_{e}\bar{\mu_{tin}}-C)\;\forall s\in \Theta ,\;i\in \Psi ,\;t\in \Phi \;n\in \Omega . \end{array}$$ (6)

### Constraints

State equation: The quantity connection between adjacent periods can be presented as a state equation. Constraint (7) defines the connection among inventory level, purchase quantity, and demand. *l*_*t*(*i*+1)_ is zero, when demand for material *t* cannot be satisfied [43].

$$\begin{array}{c} l_{t(i+1)}=l_{ti}+x_{tis}\left(1-\bar{q_{ts}}\right)-\bar{u_{ti}},\;or\;l_{t(i+1)}=0 \end{array}$$ (7)

Original and final constraints: In practical scenario, the original and final constraints can be generally set as zero.

$$\begin{array}{c} l_{t0}=S_{t}=0,\;\forall t\in \Phi , \end{array}$$ (8)

$$\begin{array}{c} l_{t(I-1)}=T_{t}=0,\;\forall t\in \Phi . \end{array}$$ (9)

Budget constraint: The manufacturers have budget constraint including purchase cost, inventory cost, transport cost, and penalty cost. Therefore, economic objective should be within the budget.

$$\begin{array}{c} F^{PC}+F^{IC}+F^{TC}+F^{PeC}\leq B\\ \forall s\in \Theta ,\;i\in \Psi ,\;t\in \Phi ,\;m\in \Pi ,\;n\in \Omega . \end{array}$$ (10)

Capacity constraints: The order amount for material *t* in each period must be within constraint (11):

$$\begin{array}{c} P_{t}^{min}\leq x_{tis}\leq P_{t}^{max},\;or\;x_{tis}=0.\;\forall s\in \Theta ,\;i\in \Psi ,\;t\in \Phi . \end{array}$$ (11)

The inventory level *l*_*ti*_ should satisfy the constraint (12):

$$\begin{array}{c} 0\leq l_{ti}\leq Q_{t}^{max}\;\forall i\in \Psi ,\;t\in \Phi . \end{array}$$ (12)

Supplier selection constraints: Every material should be purchased from one supplier, and every supplier can provide all types of materials. There should be at least one selected supplier and there should be no more selected suppliers than the material species. Therefore, the number of selected suppliers should be between one and *T*.

$$\begin{array}{c} 1\leq \sum_{s}w_{tis}\leq T\;\forall s\in \Theta ,i\in \Psi ,\;t\in \Phi . \end{array}$$ (13)

Binary variable constraint: Since the *w*_*tis*_ is binary variable, it should satisfy the constraint (14):

$$\begin{array}{c} w_{tis}=\left\{0,1\right\}\;\forall s\in \Theta ,\;i\in \Psi ,\;t\in \Phi . \end{array}$$ (14)

Non-negative constraint: Except for *w*_*tis*_, other decision variables should be non-negative.

$$\begin{array}{c} x_{tis},l_{ti},y_{tin}\geq 0\;\forall s\in \Theta ,\;i\in \Psi ,\;t\in \Phi \;n\in \Omega . \end{array}$$ (15)

## Solution method

### Normalized normal constraint method for bi-objective model

In this paper, the economic and environmental objectives are conflicting. In economic objective, if the supplier with lower price is selected, the purchase cost can be minimized, but this supplier may be far from the corresponding supply hub. According to ∑_*s*_∑_*t*_∑_*i*_ *D*_*t*_*ξ*_*c*_*ξ*_*e*_*x*_*tis*_*w*_*tis*_, the distance between supplier and supply hub has crucial influence on carbon emission. A longer distance leads to higher carbon emission as well as carbon emission cost. In the environmental objective, by contrast, supplier near the supply hub is chosen, then the carbon emission cost can be minimized, but the purchase price of the materials may be higher than the price set by other suppliers.

Under this circumstance, decision makers have to make trade-off solutions between the two objectives. The weighting, constraint, and normalized normal constraint (NNC) methods are effective to generate the Pareto solution. The NNC method was proposed by Mattson in 2003; this method presents a clear methodology for obtaining an evenly spaced Pareto solution for bi-objective problems. The core idea of the NNC method is to construct a series of single-objective optimization problems after normalized processing of the feasible region of bi-objective optimization, and then search for the Pareto solution one by one according to the direction of the Utopia line. The advantage of this method is that the evenly spaced and complete Pareto non-inferior solution can be obtained.

**Fig 3:**
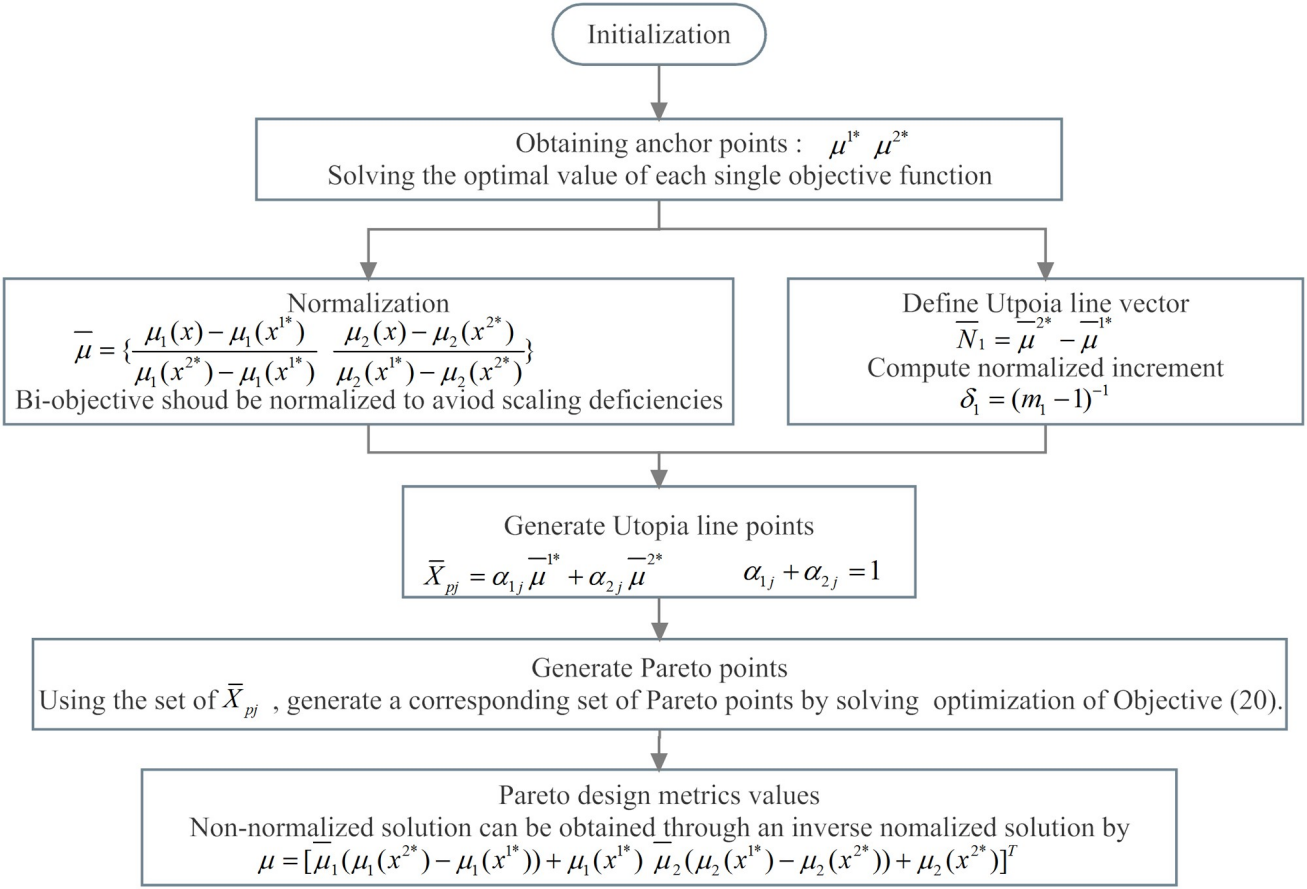
Solution procedure of NNC method.

The procedure of the of NCC method for the bi-objective model is presented in Fig 3, and the details can be found in [24]. The first step is solving the two objective functions and obtaining the two anchor points. Then, the objectives and feasible region are normalized, the utopia line vector is obtained, and the increment is normalized. After the utopia line point (${\bar{X}}_{pj}$) is generated, the bi-objective model can be transformed into two single-objective models (16) with constraints (7) (15) and additional constraint of ${\bar{N}}_{1}{\left(\bar{\mu }-{\bar{X}}_{pj}\right)}^{T}\leq 0$ and $\bar{\mu }={\left[{\bar{\mu }}_{1}\left(x\right)-{\bar{\mu }}_{2}\left(x\right)\right]}^{T}$. The Pareto points of the original bi-objective optimization problem are obtained by solving these two single-objective optimization models by utopia line points ${\bar{X}}_{pj}$.

$$\begin{array}{l} minF_{2}\\ s.t.\\ constraint(7)(15)\\ {\bar{N}}_{1}{\left(\bar{\mu }-{\bar{X}}_{pj}\right)}^{T}\leq 0\\ \bar{\mu }={\left[{\bar{\mu }}_{1}(x)\;{\bar{\mu }}_{2}(x)\right]}^{T}, \end{array}$$ (16)

The last step is calculating the value of the single-objective function for each Pareto solution, and the non-normalized design metrics can be obtained by $\mu ={\left[{\bar{\mu }}_{1}l_{1}+\mu_{1}\left(x^{1*}\right)\;{\bar{\mu }}_{2}l_{2}+\mu_{2}\left(x^{2*}\right)\right]}^{T}$.

### Differential evolution algorithm for transformed single objective model

Based on the differential evolution (DE) proposed by Storn and Price [44] in 1997, DE has been gradually applied to many fields, especially in science and engineering [45]. DE is a algorithm that uses population size *L* of *D* − *dimensional* vectors to do random search [26]. Through crossover, mutation, and selection operations, each target vector is perturbed into a trial vector. Crossover, mutation, and selection procedure are introduced in the subsequent sections.

#### Encoding and decoding strategies

To apply DE to solve IAPSSCT, the relationship between vector supplier selection and purchase quantity must be defined. The vector in encoding procedure comprises of (*s* + *t*)*i* components. The first *si* − *dimensions* of each vector describes different suppliers. The last *ti* − *dimensions* represent *t* materials and *i* periods. Each vector is decoded into the supplier selection and purchase quantities of each material in every periods in each iteration. The encoding procedure begins with initializing population size *L* of (*s* + *t*)*i* *dimensional* vectors randomly. The evolution of the population occurs mainly through the operations of mutation, crossover, and selection until the finishing criteria are met. The decoding process consists of three steps. First, a supplier selection list is constructed based on the geographical locations of suppliers. Second, the purchase quantity matrix is built. The last step is to establish the inventory level matrix, which can be realized using the state equation based on the results of the second step. The procedures of encoding and decoding strategies is presented in Table 1.

**Table 1:** Encoding and decoding procedure of IAPSSCT.

*Input*:	*i*: sets of periods; *i* ∈ Ψ = {0, 1, …, *I* − 1}	*t*: sets of materials; *t* ∈ Φ = {1, 2, …, *T*}
	*s*: sets of suppliers; *s* ∈ Θ = {1, 2, …, *S*}	*n*: sets of for manufacturers; *n* ∈ Ω = {1, 2, …, *N*}
	$\bar{\mu_{tin}}$: demand for material *t* at manufacturer *n* in the (*i* + 1)^*th*^ period $\bar{\mu_{tin}}∼N\left(\mu ,\sigma^{2}\right)$	
	$\bar{u_{ti}}$: demand for material *t* in the (*i* + 1)^*th*^ period $\bar{u_{ti}}=\sum_{n}\bar{\mu_{tin}}$	
	*x*_*tis*_: purchase quantity of material *t* from supplier *s* in the (*i* + 1)^*th*^ period	
	*w*_*tis*_: If supplier *s* is selected, then *w*_*tis*_ = 1; otherwise, *w*_*tis*_ = 0.	
*Output*:	*y*_*tin*_: allocation quantity of material *t* from supply hub *t* to manufacturer *n* in the (*i* + 1)^*th*^ period.	
	*l*_*ti*_: stock level of material *t* in the beginning of the (*i* + 1)^*th*^ period.	
*Step* 1	*y*_*tin*_ ← 0, *i* ∈ Ψ, *t* ∈ Φ *n* ∈ Ω, *l*_*ti*_ ← 0, *i* ∈ Ψ, *t* ∈ Φ	
*Step* 2	**While** *i* = 1: *I*, *t* = 1: *T* **do**	
	Generate $x_{tis}\geq \bar{u_{ti}}$, randomly.	
*Step* 2.1	The purchase amount is matched to the price discount point of supplier.	
	*w*_*tis*_ ← 1; The supplier with lowest price is selected by comparing each purchase price of the discount point.	
*Step* 2.2	Generate a distance matrix between selected suppliers and supply hubs.	
*Step* 2.3	Generate a distance matrix between between supply hubs and manufacturers.	
*Step* 2.4	Generate a defect rate ($\bar{q_{ts}}$) matrix according to selected suppliers.	
*Step* 3	Generate *y*_*tin*_ and *l*_*ti*_ based on state equation $l_{t(i+1)}=l_{ti}+x_{tis}\left(1-\bar{q_{ts}}\right)-\bar{u_{ti}}$.	
	**end**	

#### Mutation strategy

**Fig 4:**
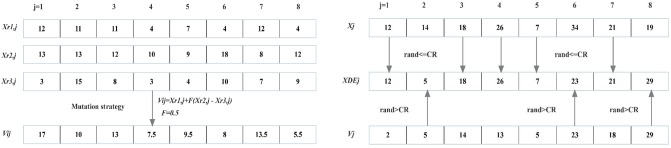
The procedures of mutation and crossover strategies. (A) The procedures of mutation strategy. (B) The procedures of crossover strategy.

The mutation procedure for target vector, *X*_*i*,*j*,*G*_, is applied to built a mutant vector, *V*_*i*,*j*,*G*_. The mutation vector of generation *G*, *V*_*i*,*j*,*G*_, are composed of three stochastically selected vectors from a present population both mutually exclusive and not the same as their related mutation vectors. A mutant vector can be created in accordance with Eq (17), where *X*_*r*1,*j*,*G*_, *X*_*r*2,*j*,*G*_, and *X*_*r*3,*j*,*G*_ are the three stochastically selected vectors. The scale factor, *F*, which can be generated based on Eqs (18) and (19), is used to scale the differential variation (*X*_*r*2,*j*,*G*_ − *X*_*r*3,*j*,*G*_) and is a parameter of DE. The procedures of crossover strategy is presented in Fig 4A.

$$\begin{array}{c} V_{i,j,G}=X_{r1,j,G}+F\left(X_{r2,j,G}-X_{r3,j,G}\right) \end{array}$$ (17)

$$\begin{array}{c} F=F_{0}\times 2\times namd \end{array}$$ (18)

$$\begin{array}{c} namd=exp(1-G_{m}/\left(G_{m}+1-G\right)). \end{array}$$ (19)

#### Crossover strategy

The trial vector, *XDE*_*i*,*j*,*G*_, is constructed by the mutant vector, *V*_*i*,*j*,*G*_, crossing with the target vector, *X*_*i*,*j*,*G*_. The diversity of perturbation parameter vectors can be enriched by crossover strategy. Exponential and binomial crossovers are the two predominatly applied crossover schemes.*CR*, defined as crossover probability, controls the part of a parameter crossed from the mutation vector in each dimension. Specifically, the trial vector, *XDE*_*i*,*j*,*G*_, is more approximate to the target vector, *X*_*i*,*j*,*G*_, when crossover probability is smaller. By contrast, the trial vector, *XDE*_*i*,*j*,*G*_, is more approximate to the mutant vector, *V*_*i*,*j*,*G*_, when crossover probability is higher. The trial vector can be generated according to Eq (20). The procedures of crossover strategy is presented in Fig 4B.

$$\begin{array}{c} XDE_{i,j,G}=\{\begin{array}{cc} & X_{i,j,G},\;if\;rand_{i,j}\leq CR\;or\;j=rand_{j}\\ & V_{i,j,G},\;if\;rand_{i,j}>CR\;or\;j\neq rand_{j}. \end{array} \end{array}$$ (20)

#### Selection strategy

The target vector of the *G* + 1 generation, *X*_*i*,*j*,*G*+1_, is constructed by selecting the target vectors, *X*_*i*,*j*,*G*+1_, or trial vector, *XDE*_*i*,*j*,*G*_, that produces a superior result compared with other methods. The target vector of the *G* + 1 generation can be generated according to Eq (21).

$$\begin{array}{c} X_{i,j,G+1}=\{\begin{array}{cc} & XDE_{i,j,G},\;if\;g\left(XDE_{i,j,G}\right)\leq g\left(X_{i,j,G}\right)\\ & X_{i,j,G},\;\;\;otherwise. \end{array} \end{array}$$ (21)

#### Overall procedure of DE

The overall procedure for DE can be described based on the preceding sections, the details of which are as follows:

*Step 1*: The parameters, *L*, *G*_0_, *G*_*m*_, *F*_0_, and *CR*, are initialized.
*Step 2*: The vectors, *X*_0_ = {*X*_1,1_, *X*_1,2_, …, *X*_1,*L*_}, are randomly initialized according to initialization strategy.
*Step 3*: The initial particles are calculated to generate the fitness value according to the transformed single objective.
*Step 4*: Three vectors, namely, *X*_*r*1,*j*,*G*_, *X*_*r*2,*j*,*G*_, and *X*_*r*3,*j*,*G*_, are selected randomly.
*Step 5*: A new mutant vector is generated based on Eq (17).
*Step 6*: A new trail vector is generated based on Eq (20).
*Step 7*: The fitness value is calculated, and the next generation of target vectors are selected based on Eq (21).
*Step 8*: If *G* = *G*_*m*_ − 1, then *G* = *G* + 1 and proceed to Step 9. Otherwise, *G* = *G* + 1 and then return to Step 4.
*Step 9*: The optimal result and optimal vector are determined.
*Step 10*: The optimal vector is decoded, and *w*_*tis*_, *x*_*tis*_ and *l*_*ti*_ (for *t* = 1, 2, …, *T*, *i* = 0, 1, 2, …, *I* − 1, *s* = 1, 2, …, *S*), are output.

## Case study

### Case presentation

To illustrate model performance and demonstrate the effect of carbon cap and carbon credit price on the environment cost, the proposed NCC-DE are applied to a case. We applied the proposed model and solution method to electronic industry with consideration of carbon emission.

**Fig 5:**
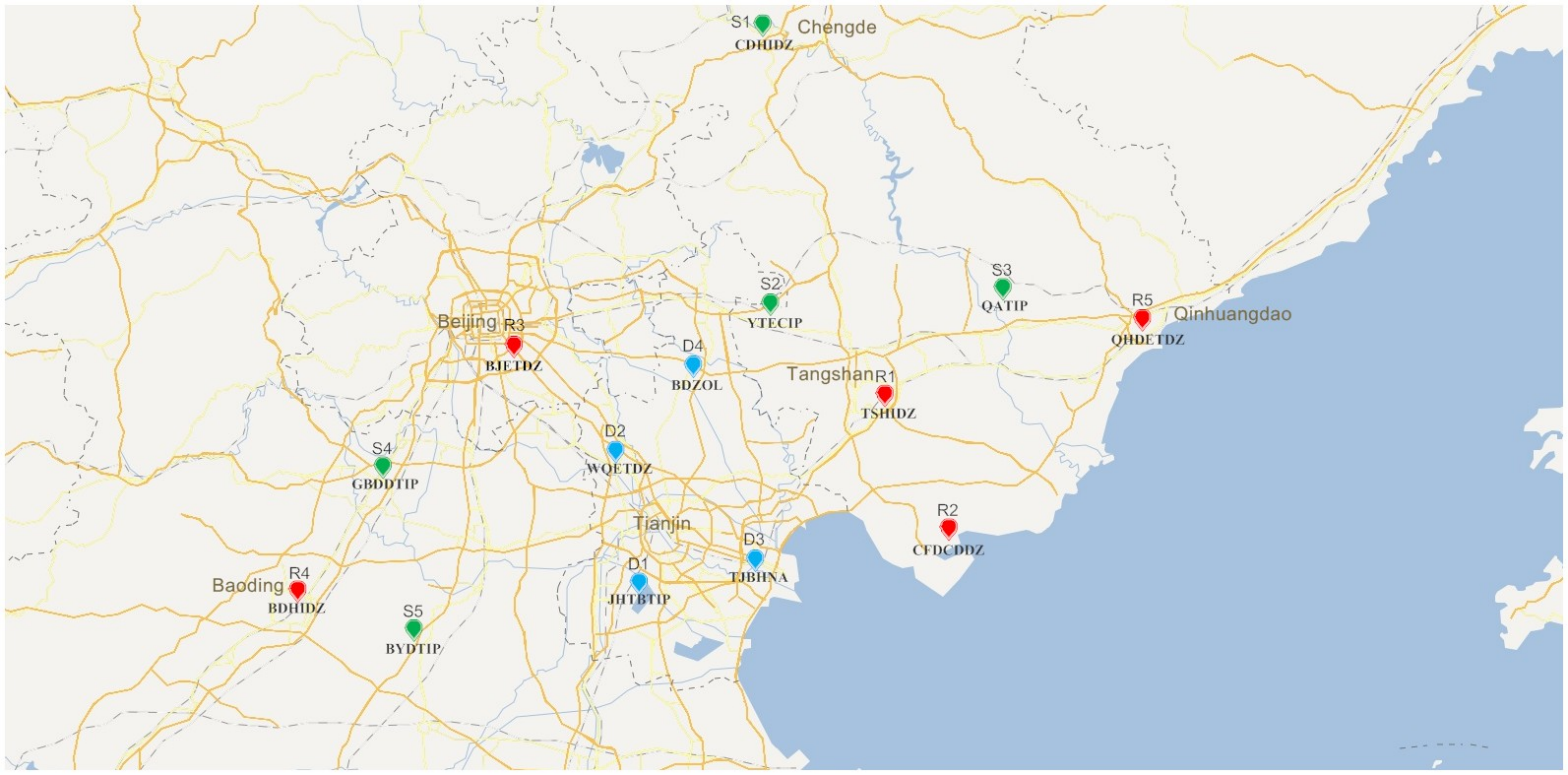
The geographic distribution of the material suppliers, supply-hubs, and electronic manufacturers.

The Beijing–Tianjin–Hebei region is an important gathering region of the electronic information industry in China. There are a number of mature industrial parks in Beijing, which have competitive advantages in attracting investment and product research and development. Tianjin has a competitive advantage in the manufacture of electronic components, integrated circuits and mobile communication equipment. The development degree of the electronic information industry in Hebei is relatively low, but its potential is huge, which creates favorable conditions for the advantageous industries to undertake the Beijing–Tianjin area. The geographic distribution of the raw material suppliers, supply-hubs, and electronic manufacturers in the Beijing–Tianjin–Hebei region is shown as Fig 5. Raw materials suppliers and electronic manufacturers are distributed in the periphery of Beijing, Tianjin, and Hebei Province, and are relatively scattered, and supply-hubs is distributed around Tianjin. Specifically, in this case, including five suppliers of raw materials: Chengde high-tech industrial development zone (CH–T), Yutian electronic components industrial park (YT–E), Gaobeidian technology industrial park (GBD-T), Qianan technology industrial park (QA–T), Baiyangdian technology industrial park (BYD–T); four supply-hubs: Jinghai Tuanbo technology industrial park (JHTB–T), Wuqing economic and technological development Zone (WQ–E), Tianjin Binhai new area (TJBH-N), Baodi ZOL (BD-Z); and five electronic manufacturer: Tangshan high-tech industrial development zone (TS-H), Caofeidian cooperation development demonstration zone (CFD–C), Beijing economic and technological development zone (BJ–E). Baoding high-tech industrial development zone (BD–H), Qinhuangdao economic and technological development zone (QHD–E).

We investigated three electronic components companies CH, CY and YT to collect relevant data needed for this study. The data was collected between May 2015 and March 2016. We also interviewed with five experts at Beijing–Tianjin–Hebei development research center and electronic information industry bodies to obtain information about transport and environmental impact on electronic information industry. Supply hubs in Beijing–Tianjin–Hebei region are the third-party business entity of inventory and allocation for manufacturers. The procurement of materials among electronic enterprises is completed by the supply hubs. The supply hub operator selected a suitable supplier for each material and decide the purchase quantity, inventory level, and allocation quantity of each material.

The present case involves five suppliers (*S*_1_, *S*_2_, *S*_3_, *S*_4_, *S*_5_), four materials (*T*_1_, *T*_2_, *T*_3_, *T*_4_), four supply-hubs (*D*_1_, *D*_2_, *D*_3_, *D*_4_) and five manufacturers (*R*_1_, *R*_2_, *R*_3_, *R*_4_, *R*_5_). Five periods are considered in the proposed supplier–manufacturer network, and every period lasts one month. The demand for each material for each month of each manufacturers is shown in Table 2. Purchase information and material inventory information are presented in Tables 3 and 4. The distribution information is provided in Table 5, and distance among suppliers, supply hubs, and manufacturers are provided in Table 6. All random parameters follows a normal distribution. Fuel consumption (*ξ*_*c*_) is 0.245 (l/km), and *CO*_2_ emissions for a unit of gasoline (*ξ*_*e*_) are 2.63 (kg/l). These emission parameters were obtained from [46].

**Table 2:** Demand information of materials.

	Month index (*i*)				
	i = 1	i = 2	i = 3	i = 4	i = 5
$\bar{u_{1i}}$	$\bar{u_{11}}∼N\left(12.82,0.77\right)$	$\bar{u_{12}}∼N\left(9.97,0.68\right)$	$\bar{u_{13}}∼N\left(11.51,0.76\right)$	$\bar{u_{14}}∼N\left(12.48,0.79\right)$	$\bar{u_{15}}∼N\left(11.60,0.78\right)$
$\bar{u_{2i}}$	$\bar{u_{2i}}∼N\left(19.40,1.23\right)$	$\bar{u_{22}}∼N\left(20.25,1.31\right)$	$\bar{u_{23}}∼N\left(22.30,1.59\right)$	$\bar{u_{24}}∼N\left(21.79,1.45\right)$	$\bar{u_{25}}∼N\left(22.59,1.76\right)$
$\bar{u_{3i}}$	$\bar{u_{31}}∼N\left(22.27,1.57\right)$	$\bar{u_{32}}∼N\left(21.74,1.48\right)$	$\bar{u_{33}}∼N\left(20.10,1.35\right)$	$\bar{u_{34}}∼N\left(22.26,1.57\right)$	$\bar{u_{35}}∼N\left(22.90,1.58\right)$
$\bar{u_{4i}}$	$\bar{u_{41}}∼N\left(29.17,2.25\right)$	$\bar{u_{42}}∼N\left(29.54,2.24\right)$	$\bar{u_{43}}∼N\left(29.67,2.26\right)$	$\bar{u_{44}}∼N\left(29.15,2.23\right)$	$\bar{u_{45}}∼N\left(31.32,2.38\right)$

**Table 3:** Purchasing information of materials.

	MAT.	Price break point	Cost	MAT.	Price break point	Cost	MAT.	Price break point	Cost	MAT.	Price break point	Cost
	10^3^	s_tm−1_ ≤ x_tis_ < s_tm_	c_tm_		s_tm−1_ ≤ x_tis_ < s_tm_	c_tm_		s_tm−1_ ≤ x_tis_ < s_tm_	c_tm_		s_tm−1_ ≤ x_tis_ < s_tm_	c_tm_
S1	T1	4.44 ≤ *x*_1*i*1_ < 5.57	2.48	T2	9.03 ≤ *x*_2*i*1_ < 10.03	0.18	T3	8.02 ≤ *x*_3*i*1_ < 9.72	0.48	T4	12.12 ≤ *x*_4*i*1_ < 13.62	0.57
		5.57 ≤ *x*_1*i*1_ < 6.70	2.45		10.03 ≤ *x*_2*i*1_ < 11.02	0.16		9.72 ≤ *x*_3*i*1_ < 11.71	0.45		13.62 ≤ *x*_4*i*1_ < 15.02	0.54
		6.7 ≤ *x*_1*i*1_ < 7.83	2.42		11.02 ≤ *x*_2*i*1_ < 12.02	0.13		11.71 ≤ *x*_3*i*1_ < 13.41	0.42		15.02 ≤ *x*_4*i*1_ < 16.52	0.51
S2	T1	4.34 ≤ *x*_1*i*2_ < 5.47	2.47	T2	9.23 ≤ *x*_2*i*2_ < 10.23	0.19	T3	8.09 ≤ *x*_3*i*2_ < 9.79	0.49	T4	12.02 ≤ *x*_4*i*2_ < 13.52	0.56
		5.47 ≤ *x*_1*i*2_ < 6.60	2.45		10.23 ≤ *x*_2*i*2_ < 11.22	0.15		9.79 ≤ *x*_3*i*2_ < 11.79	0.46		13.52 ≤ *x*_4*i*2_ < 15.12	0.53
		6.60 ≤ *x*_1*i*2_ < 7.83	2.43		11.22 ≤ *x*_2*i*2_ < 12.22	0.13		11.79 ≤ *x*_3*i*2_ < 13.49	0.44		15.12 ≤ *x*_4*i*2_ < 16.42	0.50
S3	T1	4.54 ≤ *x*_1*i*3_ < 5.67	2.48	T2	9.15 ≤ *x*_2*i*3_ < 10.15	0.19	T3	8.12 ≤ *x*_3*i*3_ < 9.82	0.48	T4	12.22 ≤ *x*_4*i*3_ < 13.72	0.56
		5.67 ≤ *x*_1*i*3_ < 6.80	2.44		10.15 ≤ *x*_2*i*3_ < 11.18	0.16		9.82 ≤ *x*_3*i*3_ < 11.81	0.46		13.72 ≤ *x*_4*i*3_ < 15.12	0.54
		6.80 ≤ *x*_1*i*3_ < 7.83	2.41		11.18 ≤ *x*_2*i*3_ < 12.22	0.11		11.81 ≤ *x*_3*i*3_ < 13.51	0.42		15.12 ≤ *x*_4*i*3_ < 16.62	0.52
S4	T1	4.24 ≤ *x*_1*i*4_ < 5.37	2.49	T2	9.19 ≤ *x*_2*i*4_ < 10.19	0.18	T3	8.22 ≤ *x*_3*i*4_ < 9.92	0.49	T4	12.19 ≤ *x*_4*i*4_ < 13.69	0.57
		5.37 ≤ *x*_1*i*4_ < 6.40	2.45		10.19 ≤ *x*_2*i*4_ < 11.19	0.14		9.92 ≤ *x*_3*i*4_ < 11.91	0.46		13.69 ≤ *x*_4*i*4_ < 15.09	0.55
		6.40 ≤ *x*_1*i*4_ < 7.53	2.42		11.19 ≤ *x*_2*i*4_ < 12.22	0.11		11.91 ≤ *x*_3*i*4_ < 13.61	0.41		15.09 ≤ *x*_4*i*4_ < 16.59	0.53
S5	T1	4.48 ≤ *x*_1*i*5_ < 5.59	2.49	T2	9.10 ≤ *x*_2*i*5_ < 10.10	0.19	T3	8.15 ≤ *x*_3*i*5_ < 9.85	0.48	T4	12.10 ≤ *x*_4*i*5_ < 13.60	0.57
		5.59 ≤ *x*_1*i*5_ < 6.78	2.47		10.10 ≤ *x*_2*i*5_ < 11.10	0.15		9.85 ≤ *x*_3*i*5_ < 11.84	0.44		13.60 ≤ *x*_4*i*5_ < 15.00	0.54
		6.78 ≤ *x*_1*i*5_ < 7.93	2.45		11.10 ≤ *x*_2*i*5_ < 12.12	0.10		11.84 ≤ *x*_3*i*5_ < 13.55	0.43		15.00 ≤ *x*_4*i*5_ < 16.60	0.51

**Table 4:** Inventory information of materials.

Material	Inspection fee	Storage cost	Return price	Penalty	Defect rate
t	d_t_ (CNY)	K_t_ (CNY)	r_t_ (CNY)	*σ*_t_(CNY)	$\bar{{\text{q}}_{\text{ts}}}\left(\%\right)$
M1	0.009	9	0.09	0.03	$\bar{q_{1s}}∼N\left(0.01788,0.71\right)$
M2	0.011	6	0.08	0.02	$\bar{q_{2s}}∼N\left(0.02112,0.88\right)$
M3	0.012	3	0.03	0.03	$\bar{q_{3s}}∼N\left(0.00876,0.91\right)$
M4	0.006	2	0.01	0.01	$\bar{q_{4s}}∼N\left(0.01812,0.65\right)$

**Table 5:** Distribution cost from supplier to supply hub.

	Month index (*i*)				
	i = 1	i = 2	i = 3	i = 4	i = 5
$\bar{\gamma_{1i}}$	$\bar{\gamma_{11}}∼N\left(21,1.4\right)$	$\bar{\gamma_{12}}∼N\left(14,0.8\right)$	$\bar{\gamma_{13}}∼N\left(15,0.9\right)$	$\bar{\gamma_{14}}∼N\left(20,1.3\right)$	$\bar{\gamma_{15}}∼N\left(15,0.9\right)$
$\bar{\gamma_{2i}}$	$\bar{\gamma_{21}}∼N\left(19,1.2\right)$	$\bar{\gamma_{22}}∼N\left(13,0.7\right)$	$\bar{\gamma_{23}}∼N\left(17,1.1\right)$	$\bar{\gamma_{24}}∼N\left(21,1.4\right)$	$\bar{\gamma_{25}}∼N\left(17,1.1\right)$
$\bar{\gamma_{3i}}$	$\bar{\gamma_{31}}∼N\left(21,1.4\right)$	$\bar{\gamma_{32}}∼N\left(14,0.8\right)$	$\bar{\gamma_{33}}∼N\left(15,0.9\right)$	$\bar{\gamma_{34}}∼N\left(20,1.3\right)$	$\bar{\gamma_{35}}∼N\left(15,0.9\right)$
$\bar{\gamma_{4i}}$	$\bar{\gamma_{41}}∼N\left(19,1.2\right)$	$\bar{\gamma_{42}}∼N\left(23,1.7\right)$	$\bar{\gamma_{43}}∼N\left(27,2.1\right)$	$\bar{\gamma_{44}}∼N\left(31,2.3\right)$	$\bar{\gamma_{45}}∼N\left(27,2.1\right)$

**Table 6:** Distance among suppliers, supply hubs, and retail stores (km).

Supply hub *t* & MFR *n*	MFR 1	MFR 2	MFR 3	MFR 4	MFR 5	Supplier 1	Supplier 2	Supplier 3	Supplier 4	Supplier 5
Supply hub 1 (D1)	189.2	180.9	124.6	169.6	304.9	244.9	171.1	257.2	146.9	106.1
Supply hub 2 (D2)	139.4	155.6	85.6	188.5	270.9	186.5	124.2	207.4	123.1	210.1
Supply hub 3 (D3)	170.5	98.8	162.7	244.2	210.3	225.4	142.8	175.3	204.5	221.7
Supply hub 4 (D4)	126.0	169.9	88.1	232.3	206.9	111.1	43.6	133.9	173.8	266.5

### Parameter selection using the Taguchi method

One of the important stages in designing a hybrid algorithm is to adjust the parameters that can affect the effectiveness of the algorithm. While the adjusting process is hard to perform manually, the Taguchi experiments is conducted for this purpose. In the Taguchi method, the factors are divided into two categories: controllable factors and noise factors. Based on the concept of robustness, the Taguchi method tries to minimize the impact of noise factors and determine the optimal level of important controllable factors. Taguchi method developed signal–to–noise (*S*/*N*) ratio to evaluate whether this type of parameters is robust. In this research the parameters of the algorithm includes *G*_*m*_ (iteration), *L* (population size), *F* (scale factor), and *CR* (crossover probability). The set levels for the algorithm parameters are described in Table 7. The L9(3**3) design in the MINITAB is used for adjusting the parameters. The Taguchi experimental results are presented in Table 8.

**Table 7:** The levels for each of the parameters.

Level of factor	*L*	*G*_*m*_	*F*_0_	*CR*
1	15	200	0.5	0.8
2	20	250	0.6	0.9
3	25	300	0.7	1.0

**Table 8:** Normalized results from the Taguchi experiments.

Exp.No.	*L*	*G*_*m*_	*F*_0_	*CR*	*rep*1	*rep*2	*rep*3	*rep*4	*rep*5
1	15	200	0.5	0.8	3543254	3428129	3487698	3540597	3418757
2	15	250	0.6	0.9	3760987	3750393	3591354	3657725	3676522
3	15	300	0.7	1.0	3570690	3637331	3637331	3524167	3654849
4	20	200	0.6	1.0	3542194	3616531	3646553	3655497	3639408
5	20	250	0.7	0.8	3623913	3690123	3667825	3524319	3666743
6	20	300	0.5	0.9	3542069	3527126	3658842	3554336	3698765
7	25	200	0.7	0.9	3570042	3543272	3612589	3583760	3650989
8	25	250	0.5	1.0	3511213	3439684	3591349	3445364	3454658
9	25	300	0.6	0.8	3672135	3625639	3528508	3543276	3645697

**Fig 6:**
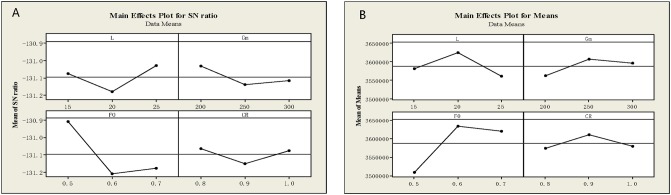
Results of Taguchi experiments. (A) SNR graph from the Taguchi experiments. (B) Mean graph from Taguchi experiments.

According to the Fig 6A the maximum *S*/*N* ratio have occurred for the *L* parameter in level 3, for the *G*_*m*_ parameter in level 1, for the *F* parameter in level 1, and for *CR* parameter in level 1. In the Fig 6B, the minimum Mean appeared at the maximum *S*/*N* ratio. Thus, the algorithm parameters can be adjusted as: *L* = 25 *G*_*m*_ = 200 *F*_0_ = 0.5 *CR* = 0.8.

### Result analysis

**Fig 7:**
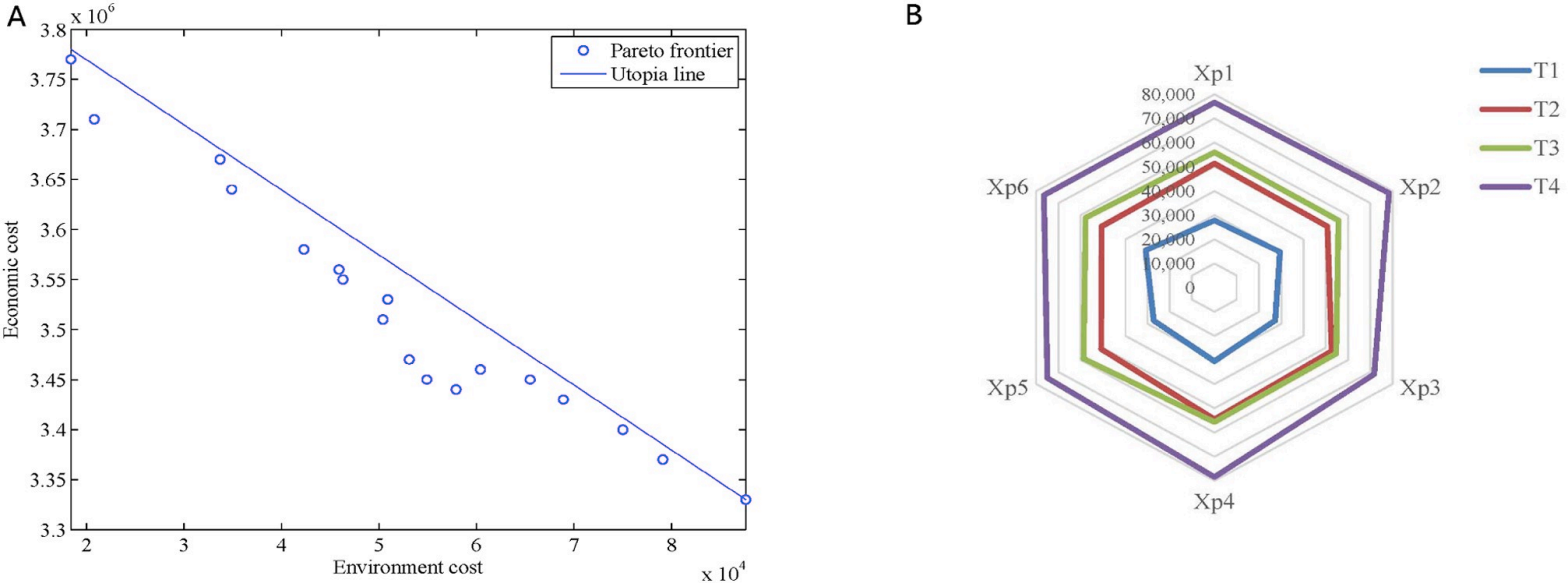
Simulation result. (A) Pareto frontier of economic and environmental objectives. (B) Purchase quantities of materials with different *X*_*pj*_.

**Fig 8:**
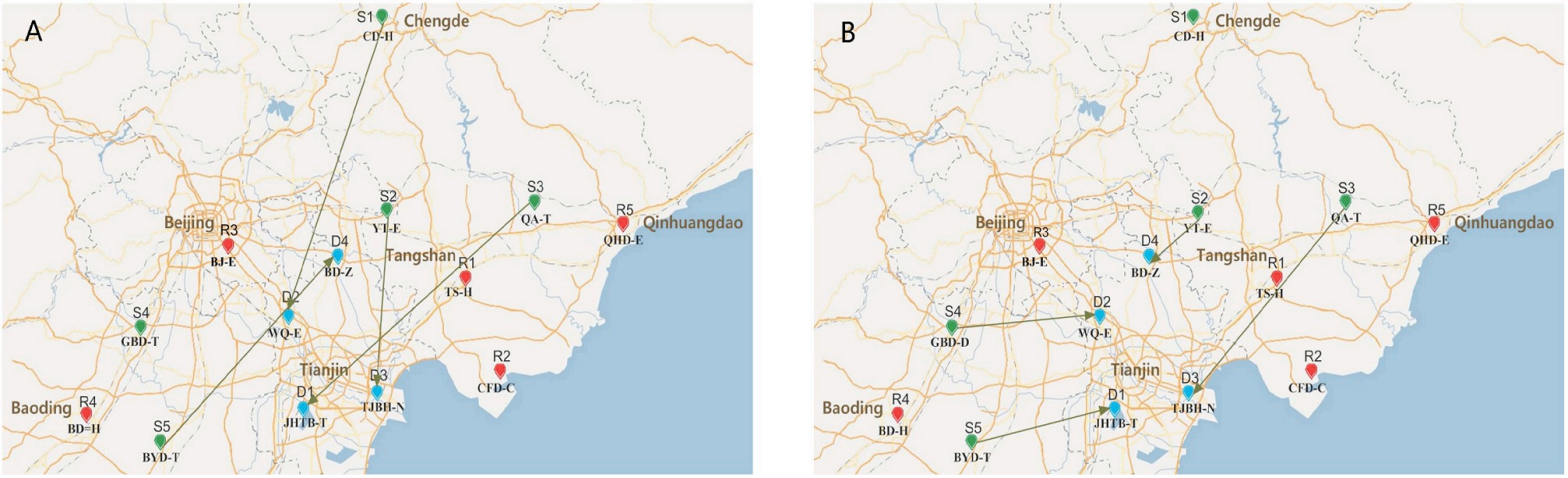
Supplier selection under different situation. (A) Supplier selection under economic condition. (B) Supplier selection under environmental condition.

The experiments represented in this section were conducted using MATLAB R2014a on a Core i5-5200U, 2.19 GHz clock pulse with 3.88 GB memory, with the aforementioned data to test the performance of the proposed mathematical model and solution method. The result in Table 9, Figs 7A, 7B, 8A and 8B are obtained via simulation for 30 times (the carbon cap is 3000 tons, and the carbon credit price (*ϖ*) is 36.29 CNY/ton in Tianjin in 2016).

**Table 9:** Typical Pareto solutions of inventory allocation planning problem.

Solutions	Optimal order quantity											Economic	Environmental
**1**	**Variable**	**Month index**										3.72 × 10^6^	2.14 × 10^4^
		**i** = **0**		**i** = **1**		**i** = **2**		**i** = **3**		**i** = **4**			
	T1	7359.2	S3	5282.3	S3	6794.8	S3	7361.5	S4	4417.3	S4		
	T2	11257.3	S1	13994.9	S4	9480.9	S1	11144.5	S5	13994.9	S1		
	T3	14351.8	S4	11596.6	S3	9010.1	S5	9150.8	S5	9264.0	S3		
	T4	16521.0	S3	17198.9	S5	13994.9	S3	11780.9	S3	13047.8	S5		
**2**	**Variable**	**Month index**										3.62 × 10^6^	3.39 × 10^4^
		**i** = **0**		**i** = **1**		**i** = **2**		**i** = **3**		**i** = **4**			
	T1	8754.6	S4	5874.2	S3	6984.2	S4	9945.1	S3	7787.3	S3		
	T2	12321.1	S1	10025.6	S1	9998.6	S4	13658.6	S5	13445.9	S5		
	T3	13658.2	S3	12663.1	S2	10025.6	S2	10037.9	S4	9587.8	S3		
	T4	15464.9	S1	14702.1	S5	10998.5	S5	16653.1	S3	15584.2	S3		
**3**	**Variable**	**Month index**										3.54 × 10^6^	4.53 × 10^4^
		**i** = **0**		**i** = **1**		**i** = **2**		**i** = **3**		**i** = **4**			
	T1	6587.4	S3	8598.7	S2	5569.1	S3	7851.3	S2	4986.2	S3		
	T2	12258.3	S4	14002.1	S2	13692.7	S4	12988.8	S5	10920.0	S2		
	T3	11316.4	S3	13211.5	S3	10010.2	S2	13601.3	S3	8475.9	S2		
	T4	12214.3	S4	16658.1	S3	14758.9	S5	15552.4	S3	10101.3	S5		
**4**	**Variable**	**Month index**										3.52 × 10^6^	5.01 × 10^4^
		**i** = **0**		**i** = **1**		**i** = **2**		**i** = **3**		**i** = **4**			
	T1	4989.9	S4	5698.1	S4	6483.9	S3	9455.1	S3	7365.2	S4		
	T2	10025.2	S5	14070.9	S4	11013.7	S1	10009.8	S5	12225.5	S5		
	T3	14142.3	S3	11259.8	S2	10025.3	S5	13986.5	S5	10005.5	S2		
	T4	16665.3	S3	14573.2	S5	12202.0	S2	16652.4	S5	13254.9	S4		
**5**	**Variable**	**Month index**										3.47 × 10^6^	5.67 × 10^4^
		**i** = **0**		**i** = **1**		**i** = **2**		**i** = **3**		**i** = **4**			
	T1	7125.8	S2	8211.1	S3	4699.0	S3	6854.1	S2	7700.1	S3		
	T2	16748.5	S4	12252.2	S2	13337.5	S5	13015.8	S2	10210.4	S4		
	T3	11258.3	S2	10057.6	S3	12412.7	S5	8547.9	S3	9943.1	S2		
	T4	11142.9	S2	13528.6	S5	14187.5	S2	12003.8	S4	10077.9	S4		
**6**	**Variable**	**Month index**										3.43 × 10^6^	7.30 × 10^4^
		**i** = **0**		**i** = **1**		**i** = **2**		**i** = **3**		**i** = **4**			
	T1	7415.8	S4	5465.1	S4	6653.2	S5	7124.5	S4	5599.8	S4		
	T2	11214.7	S2	12568.0	S2	13697.3	S4	15001.2	S4	11254.3	S4		
	T3	14142.8	S5	8872.5	S2	10098.4	S3	12197.6	S2	10014.5	S5		
	T4	13363.5	S2	14002.8	S1	14254.3	S2	12225.7	S4	10091.7	S1		
**7**	**Variable**	**Month index**										3.36 × 10^6^	8.51 × 10^4^
		**i** = **0**		**i** = **1**		**i** = **2**		**i** = **3**		**i** = **4**			
	T1	6893.1	S5	6579.2	S4	6565.0	S5	7700.4	S5	4491.5	S5		
	T2	13684.0	S4	12945.2	S2	11942.6	S4	13205.3	S4	11024.3	S4		
	T3	13567.2	S3	11464.8	S5	12681.9	S2	13821.8	S3	11623.5	S3		
	T4	16571.0	S2	14681.9	S4	15773.9	S4	15798.2	S1	14541.1	S2		

Inventory and allocation decisions with carbon trading across multiple periods are critical for IAPSSCT. Fig 7A illustrates the Pareto frontier of economic and environmental objectives. The intersection of the utopia line and *X* axis is the optimal solution of environmental objective, and the intersection of the utopia line and *Y* axis is the optimal solution of economic objective (the two intersections are also the anchor point in the NNC method). As mentioned in the previous chapter, the utopia line points *X*_*pj*_ are a set of evenly distributed points, and the Pareto frontier can be generated by optimizing the single objective with constraint ${\bar{N}}_{1}{\left(\bar{\mu }-{\bar{X}}_{pj}\right)}^{T}\leq 0$. A spider chart illustrates the floating of purchase quantities with different *X*_*pj*_ in the five periods. As shown in Fig 7B, with changes in *X*_*pj*_, the purchase quantities of the four materials have changed slightly. This condition indicates that the purchase quantities are not the critical influence factor of the Pareto non-inferiority solution of IAPSSCT.

In order to further observe the strategies under the economic and environmental objectives, the typical solutions and specific purchase and supplier selection strategies on the Pareto frontier are given as shown in Table 9. Although the purchase quantities of these four materials are similar in each period, whereas the selections of suppliers vary. A conflict occurs between the economic and environmental objectives. When it comes to the minimization of economic objective, the decision maker prefers to select the supplier with low purchase price, which may lead to long distance transport and high environmental cost, and vice versa. T1 in the first period is used as an example. The economic cost of selecting supplier S3 is the lowest, as shown in Fig 8A. In the case of carbon emissions, the decision maker selects S5, as shown in Fig 8B, which is closer to D1, even if the purchase price is higher, to reduce carbon emissions during transportation, cutting carbon emissions and costs or earning carbon credits. Solution 1 is the optimal result of minimizing the cost of the economy without considering carbon emissions. Table 9 shows that a larger reduction in carbon emissions can be achieved with smaller economic costs after taking into account the carbon trading. Compared with the traditional single economic cost model, considering carbon trading has better flexibility and better adaptability to the change of carbon emissions policies.

#### Carbon emission analysis

**Fig 9:**
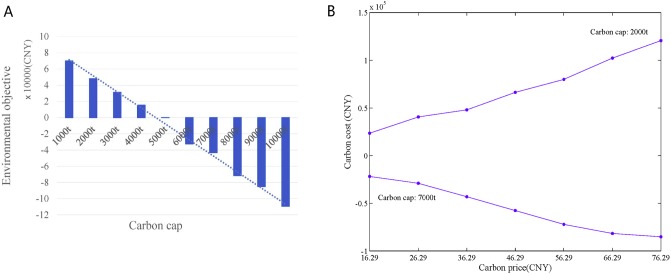
Impact of carbon emission. (A) Environmental cost under different carbon caps. (B) Floating of environmental cost with different carbon credit prices.

The carbon price in the carbon trading market of Tianjin is 36.29 CNY/ton. With an increase in the carbon cap, environmental objective experience a downward trend with the same carbon credit price, as shown in Fig 9A. When the carbon cap increases from 1000 t to 5000 t, the carbon costs reduced from 6.983 × 10^4^ (CNY) to 96.574 (CNY). When the carbon cap increases from 6000 t to 10000 t, carbon costs continue to decrease to a negative value. The Kyoto Protocol mandates that companies which do not reach their carbon emission cap can sell the excess to other companies. In such a case, the carbon costs of a company will begin to decline toward a negative value, at which point, the company will earn credits. In other words, a negative cost shows that a company is indeed profiting by diminishing its carbon emissions. Furthermore, the carbon cap is conducive to economic growth. However, as the environment becomes increasingly damaged, the carbon cap will be tightened, which will eventually cause a negative economic effect. In fact, from perspective of enterprises, selecting the best decision based on the preferences of decision makers under varying carbon caps is appropriately given that the carbon cap exhibits strong externality.

When carbon emissions are higher than the carbon cap threshold (denoted as: *Cap*^*TH*^, which is approximately 5000 t in this case), in which carbon cost increases with an increase in carbon credit price (for example, carbon cap = 2000 t). When carbon emissions are below *Cap*^*TH*^ (for example, carbon cap = 7000 t), environmental objective decrease with an increase in carbon credit price, as these show in Fig 9B.

**Fig 10:**
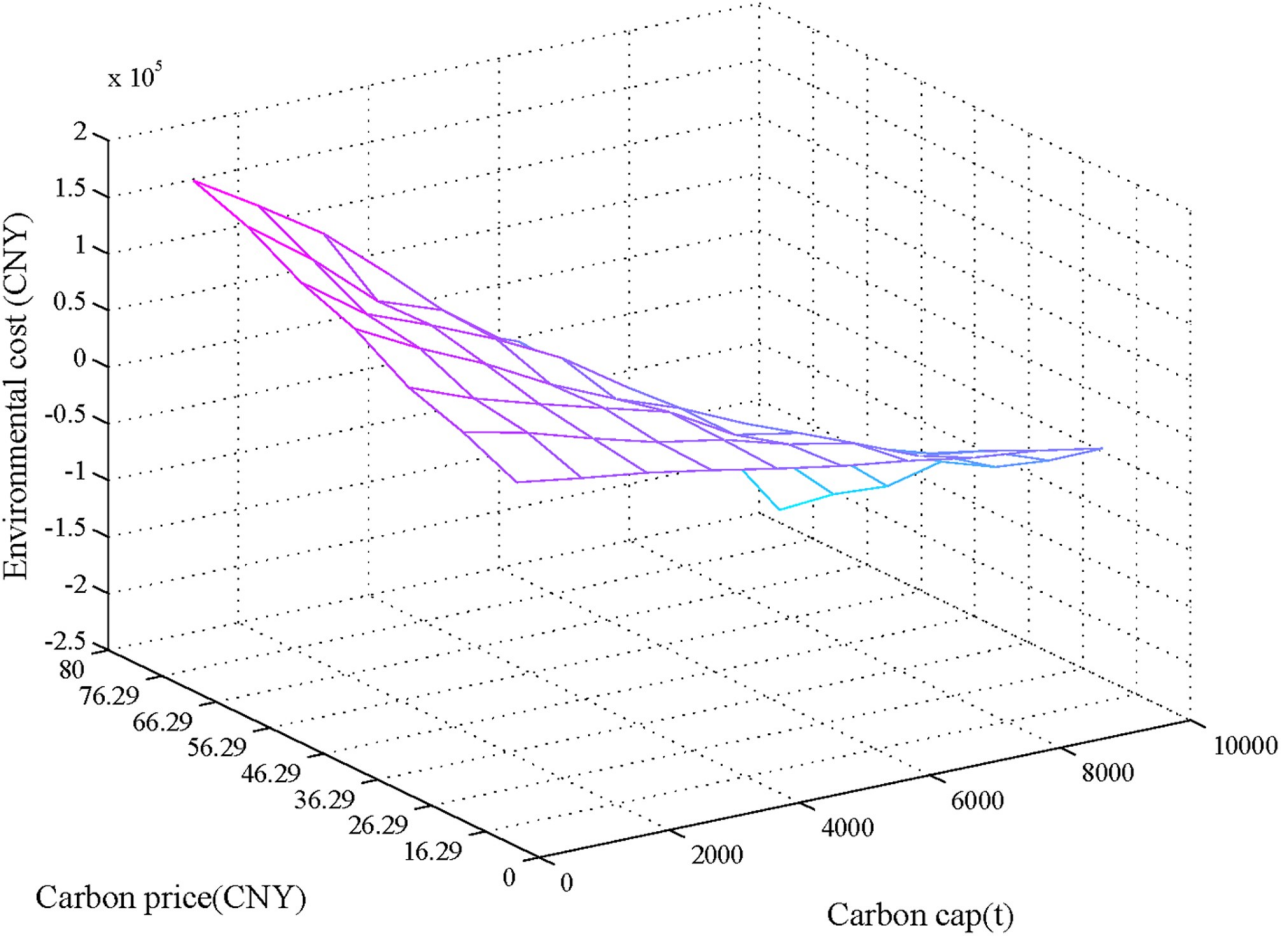
Impact of carbon cap and carbon credit prices on environmental objective.

After simulation for 30 times, Fig 10 shows the statistical results of the carbon emissions costs with the carbon cap ranging from 1000 t to 10000 t, and the carbon credit price ranging from 16.29 (CNY/t) to 76.29 (CNY/t). With the international carbon trading, and carbon emission reduction pressure gradually increasing, the carbon cap is gradually reduced, and the credit price of carbon trading increases. The carbon credit price becomes close to international carbon credit price (7.35 (EUR/t), or approximately 66.29 (CNY/t)). The impacts of the carbon cap and the carbon credit prices on environmental costs, as shown in Fig 10, are consistent and exhibit a reduction as the carbon cap increases and the carbon credit price decrease.

#### Results of large scale problems

In this network, purchasing quantities become large and the distribution network becomes more complex when the variety of materials increases. As shown in Table 10, the economic cost and environmental cost with bigger data scale are incremental. This calculation make decision makers to recognize the importance of environmental cost and reduce carbon emissions as much as possible. An obvious result can be seen from the 50 × 16 × 80 and 80 × 20 × 80 cases. Although there is an increase in the economic cost, the increase in environmental cost in the 50 × 16 × 80 case is almost the same as in the 80 × 20 × 80 case. This can be attributed to the fact that when the variety of materials and supply hubs have increased, the overall transport costs increase, while carbon emission reduces because long–distance transport decrease. In order to explore the impact of the quantity of manufacturers on economic and environmental costs, we have conduct the simulation with a fixed size 10 × 4, 20 × 8, 30 × 12, 50 × 16, and 80 × 20, while changing the numbers of manufacturers. The results indicated that the environmental costs increased significantly, while the manufacturers’ quantities increase.

**Table 10:** Simulation results of large scale problem.

No.	Parameters			Carbon price	Carbon cap	Economic cost (CNY)	Environmental cost
	*s*	*t*	*n*	(CNY)	(t)	(CNY)	(CNY)
1	10	4	10	36.29	2000	7.727 × 10^6^	3.236 × 10^5^
2	10	4	20	36.29	2000	1.601 × 10^7^	4.688 × 10^5^
3	10	4	30	36.29	2000	2.530 × 10^7^	8.699 × 10^5^
4	20	8	20	36.29	2000	3.055 × 10^7^	4.562 × 10^5^
5	20	8	30	36.29	2000	4.985 × 10^7^	6.776 × 10^5^
6	20	8	50	36.29	2000	8.292 × 10^7^	1.253 × 10^6^
7	30	12	30	36.29	2000	7.378 × 10^7^	6.101 × 10^5^
8	30	12	50	36.29	2000	1.203 × 10^8^	1.261 × 10^6^
9	30	12	80	36.29	2000	1.897 × 10^8^	1.806 × 10^6^
10	50	16	50	36.29	2000	1.638 × 10^8^	1.208 × 10^6^
11	50	16	80	36.29	2000	2.595 × 10^8^	1.575 × 10^6^
12	50	16	100	36.29	2000	3.268 × 10^8^	2.169 × 10^6^
13	80	20	80	36.29	2000	3.235 × 10^8^	1.585 × 10^6^
14	80	20	100	36.29	2000	3.884 × 10^8^	1.876 × 10^6^
15	80	20	120	36.29	2000	4.735 × 10^8^	2.429 × 10^6^

#### Algorithm comparison

In order to better illustrate the effectiveness of the proposed NNC–DE algorithm, we present the results between the NNC–PSO, and the NNC–DE algorithm. In this network, fifteen instances test data validate the proposed algorithm considering the large scale for our research problem. The weighing method, the NNC–PSO, and the NNC–DE were run 30 times with the same data. The population size and maximize iteration for all algorithms were set as: *L* = 25 and *G*_*m*_ = 200. In NNC–PSO, the acceleration constant was designed as *c*_*l*_ = *c*_*n*_ = 2 and the inertia weight was *ω*(1) = 1 and *ω*(*T*) = 0.1. The comparisons of the performance of the algorithm are presented in Table 11.

**Table 11:** Algorithm comparison.

No.	Parameters			NNC–PSO			NNC–DE		
	*s*	*t*	*n*	CPU (s)	Obj1 (CNY)	Obj2 (CNY)	CPU (s)	Obj1 (CNY)	Obj2 (CNY)
1	10	4	10	54.536	7.992 × 10^6^	3.362 × 10^5^	66.370	7.727 × 10^6^	3.236 × 10^5^
2	10	4	20	93.837	1.663 × 10^7^	4.924 × 10^5^	119.154	1.601 × 10^7^	4.688 × 10^5^
3	10	4	30	176.531	2.942 × 10^7^	8.808 × 10^5^	189.441	2.530 × 10^7^	8.699 × 10^5^
4	20	8	20	200.332	4.245 × 10^7^	5.876 × 10^5^	230.713	3.055 × 10^7^	4.562 × 10^5^
5	20	8	30	442.594	5.328 × 10^7^	7.901 × 10^5^	583.778	4.985 × 10^7^	6.776 × 10^5^
6	20	8	50	594.284	1.025 × 10^8^	1.436 × 10^6^	689.557	8.292 × 10^7^	1.253 × 10^6^
7	30	12	30	811.724	9.157 × 10^8^	8.243 × 10^5^	1068.734	7.378 × 10^7^	6.101 × 10^5^
8	30	12	50	1005.862	1.339 × 10^8^	1.522 × 10^6^	1364.878	1.203 × 10^8^	1.261 × 10^6^
9	30	12	80	1175.153	2.216 × 10^8^	2.179 × 10^6^	1443.911	1.897 × 10^8^	1.806 × 10^6^
10	50	16	50	1342.042	1.985 × 10^8^	1.674 × 10^6^	1542.887	1.638 × 10^8^	1.208 × 10^6^
11	50	16	80	1598.464	2.753 × 10^8^	2.042 × 10^6^	1647.905	2.595 × 10^8^	1.575 × 10^6^
12	50	16	100	1803.769	4.035 × 10^8^	2.661 × 10^6^	1939.873	3.268 × 10^8^	2.169 × 10^6^
13	80	20	80	1936.243	3.692 × 10^8^	2.294 × 10^6^	2187.545	3.235 × 10^8^	1.585 × 10^6^
14	80	20	100	2434.153	4.321 × 10^8^	2.637 × 10^6^	2613.234	3.884 × 10^8^	1.876 × 10^6^
15	80	20	120	3324.725	9.919 × 10^8^	6.982 × 10^6^	3717.753	4.735 × 10^8^	2.429 × 10^6^

From the Table 11, we can see the NNC–DE plays the better performance than NNC–PSO, and obtain better solution. Although NNC–DE requires more CPU time than NNC–PSO, its computing time is acceptable. With the expansion of the scale, the NNC–DE stay stable computing the optimal result. When the network size extent to 80 × 20 × 120, the output of NNC–PSO seems to be abnormal, may be trapped in a local optimal solution. Therefore, the NNE–DE algorithm is effective and efficient for extensive logistic network.

#### Managerial insight

Some managerial insights can be obtained. First, environmental factors can significantly affect the inventory allocation system, particularly when the network dimension is large or carbon emissions are strictly limited. This indicates that a supplier–manufacturer model considering the carbon cap and carbon credit price should be constructed, so that an optimal strategy can be provided to the decision makers. Therefore, companies are recommended to work for sustainable inventory allocation networks given the existence of a global movement toward the reduction of carbon emissions. Second, as energy prices rising and carbon policy tightening, we should make a balance between the purchasing cost and the carbon emission when choosing a proper supplier. Because the high carbon emission may leads to high environmental cost, while the total cost may be higher than other choices. Third, carbon emission analysis could help policy makers adjust carbon policy.

## Conclusion

In this study, a bi-objective inventory allocation planning model with supplier selection and carbon trading under uncertain environment was presented, which is proposed to find the trade-off between economic and environmental objectives by determining supplier selection, purchase quantity, inventory quantity and allocation quantity. The economic objective of this model consisted of purchase costs, inventory costs, transport costs, penalty costs, and the environmental objective was to minimize carbon emissions costs. In the proposed model, inventory and allocation planning under uncertainty was considered. Demands of manufacturers, transport price, and defect rate of materials were regarded as random variables. The model of this study extended previous findings, most of which assumed no defective materials in the purchase process. Considering the complexity of the model, a combined solution algorithm, NNC–DE, was proposed to generate the Pareto frontier of IAPSSCT. Parameter selection and algorithm comparison demonstrated the efficiency and effectiveness of the proposed NNC-DE. A case study was presented to analyze decision makers’ strategies and the impacts of a carbon cap and carbon credit price on two objectives. The decision maker can select the appropriate solution in a non-inferior set based on preference and select different strategies under different external conditions. Analysis results shows that when considering carbon cap and carbon trading, the purchase quantities, inventory level and allocation quantities are similar in each period, whereas the selections of suppliers differ remarkably. We extended the results of numerical examples in large scale problems, and compared the NNC–DE method with NNC–PSO method. The impacts of the carbon cap and carbon credit prices on the environmental costs are consistent and exhibit a reduction as the carbon cap increases and the carbon credit prices decrease.

This study expanded existing research on carbon emission in supply chains and paved the way for the development and implementation of low-carbon inventory allocation networks, which could guide managers to efficiently evaluate sustainable practices in their supplier–manufacturer networks. This framework can assist managers in simultaneously achieving economic growth and environmental protection. The proposed model can be extended by considering recycling processes and addressing increasingly strict scenarios for carbon footprint control.

## Supporting information

S1 File Details of input parameters of test instance. (DOCX)
