# Supplementary material for: Bi-objective inventory allocation planning problem with supplier selection and carbon trading under uncertainty
Source: PLoS One. 2018 Nov 28;13(11):e0206282. doi: 10.1371/journal.pone.0206282 (PMC6261456; doi:10.1371/journal.pone.0206282)
Supplement: S1 File — (DOCX) [file pone.0206282.s001.docx]

**Appendixes**

**Details of input parameters of test instance are shown as Table 1-5**

Table 1. Demand information of materials

|  | Month index ($i$ $i$) | | | | |
| --- | --- | --- | --- | --- | --- |
|  | $i=1$ | $i=2$ | $i=3$ | $i=4$ | $i=5$ |
| $\bar{u_{1i}}$ | $\theta_{11}$ $\bar{u_{11}}$~N(12.82,0.77) | $\theta_{12}$ $\bar{u_{12}}$~N(9.97,0.68) | $\theta_{13}$ $\bar{u_{13}}$~N(11.51,0.76) | $\theta_{21}$ $\bar{u_{14}}$~N(12.48,0.79) | $\theta_{22}$ $\bar{u_{15}}$~N(11.60,0.78) |
| $\bar{u_{2i}}$ | $\theta_{11}$ $\bar{u_{21}}$~N(19.40,1.23) | $\theta_{12}$ $\bar{u_{22}}$~N(20.10,1.35) | $\bar{u_{23}}$ $\theta_{13}$~N(22.30,1.59) | $\bar{u_{24}}$ $\theta_{14}$~N(21.79,1.45) | $\bar{u_{25}}$ $\theta_{21}$~N(11.60,0.78) |
| $\bar{u_{3i}}$ | $\bar{u_{31}}$ $\theta_{22}$~N(22.27,1.57) | $\bar{u_{32}}$ $\theta_{23}$~N(21.74,1.48) | $\bar{u_{33}}$ $\theta_{24}$~N(20.10,1.35) | $\bar{u_{34}}$ $\theta_{31}$∼N(29.15,2.23) | $\bar{u_{35}}$ $\theta_{32}$∼N(22.90,1.58) |
| $\bar{u_{4i}}$ | $\bar{u_{41}}$ $\theta_{33}$∼N(29.17,2.25) | $\bar{u_{42}}$ $\theta_{34}$∼N(29.54,2.24) | $\bar{u_{43}}$ $\theta_{33}$∼N(20.10,1.35) | $\bar{u_{44}}$ $\theta_{34}$∼N(29.15,2.23) | $\bar{u_{45}}$ $\theta_{23}$~N(31.32,2.38) |

Table 2. Purchasing information of materials

|  | MAT  ${10}^{3}$ | Price break point | Cost | MAT | Price break point | Cost | MAT | Price break point | Cost | MAT | Price break point | Cost |
| --- | --- | --- | --- | --- | --- | --- | --- | --- | --- | --- | --- | --- |
|  |  | $s_{tm-1}\leq x_{tis}\leq s_{tm}$ | $c_{tm}$ |  | $s_{tm-1}\leq x_{tis}\leq s_{tm}$ | $c_{tm}$ |  | $s_{tm-1}\leq x_{tis}\leq s_{tm}$ | $c_{tm}$ |  | $s_{tm-1}\leq x_{tis}\leq s_{tm}$ | $c_{tm}$ |
| $S_{1}$ | $T_{1}$ | 4.44 $\leq x_{1i1}<$5.57 $\leq x_{1i1}$ | 2.48 | $T_{2}$ | 9.03$\leq x_{2i1}<$10.03 | 0.18 | $T_{3}$ | 8.02 $\leq x_{3i1}<$9.72$\leq x_{1i1}$ | 0.48 | $T_{4}$ | 12.12$\leq x_{4i1}<$13.62 | 0.57 |
|  |  | 5.57 $\leq x_{1i1}<$6.70 | 2.45 |  | 10.03$\leq x_{2i1}<$11.02 | 0.16 |  | 9.72 $\leq x_{3i1}<$11.71 | 0.45 |  | 13.62$\leq x_{4i1}<$15.02 | 0.54 |
|  |  | 6.70 $\leq x_{1i1}$ 7.83 | 2.42 |  | 11.02$\leq x_{2i1}<$12.02 | 0.13 |  | 11.71$\leq x_{3i1}<$13.41 | 0.42 |  | 15.02$\leq x_{4i1}<$16.52 | 0.51 |
| $S_{2}$ | $T_{1}$ | 4.34$\leq x_{1i2}<$5.47 | 2.47 | $T_{2}$ | 9.23$\leq x_{2i2}<$10.23 | 0.19 | $T_{3}$ | 8.09 $\leq x_{3i2}<$ 9.79 | 0.49 | $T_{4}$ | 12.02$\leq x_{4i2}<$13.52 | 0.56 |
|  |  | 5.47 $\leq x_{1i2}<$6.60 | 2.45 |  | 10.23$\leq x_{2i2}<$11.22 | 0.15 |  | 9.79 $\leq x_{3i2}<$11.79 | 0.46 |  | 13.52$\leq x_{4i2}<$15.12 | 0.53 |
|  |  | 6.60$\leq x_{1i2}<$7.83 | 2.43 |  | 11.22$\leq x_{2i2}<$12.22 | 0.13 |  | 11.79$\leq x_{3i2}<$13.49 | 0.44 |  | 15.12$\leq x_{4i2}<$16.42 | 0.50 |
| $S_{3}$ | $T_{1}$ | 4.54 $\leq x_{1i3}<$5.67 | 2.48 | $T_{2}$ | 9.15$\leq x_{2i3}<$10.15 | 0.19 | $T_{3}$ | 8.12$\leq x_{3i3}<$ 9.92 | 0.48 | $T_{4}$ | 12.22$\leq x_{4i3}<$13.72 | 0.56 |
|  |  | 5.67$\leq x_{1i3}<$6.80 | 2.44 |  | 10.15$\leq x_{2i3}<$11.18 | 0.16 |  | 9.92$\leq x_{3i3}<$ 11.91 | 0.46 |  | 13.72$\leq x_{4i3}<$15.12 | 0.54 |
|  |  | 6.80$\leq x_{1i3}<$7.83 | 2.41 |  | 11.18$\leq x_{2i3}<$12.22 | 0.11 |  | 11.91$\leq x_{3i3}<$13.61 | 0.42 |  | 15.12$\leq x_{4i3}<$16.62 | 0.52 |
| $S_{4}$ | $T_{1}$ | 4.24 $\leq x_{1i4}<$5.37 | 2.49 | $T_{2}$ | 9.19$\leq x_{2i4}<$10.19 | 0.18 | $T_{3}$ | 8.22 $\leq x_{3i4}<$ 9.92 | 0.49 | $T_{4}$ | 12.19$\leq x_{4i4}<$13.69 | 0.57 |
|  |  | 5.37 $\leq x_{1i4}<$6.40 | 2.45 |  | 10.19$\leq x_{2i4}<$11.19 | 0.14 |  | 9.92 $\leq x_{3i4}<$11.91 | 0.46 |  | 13.69$\leq x_{4i4}<$15.09 | 0.55 |
|  |  | 6.40$\leq x_{1i4}<$7.53 | 2.42 |  | 11.19$\leq x_{2i4}<$12.22 | 0.11 |  | 11.91$\leq x_{3i4}<$13.61 | 0.41 |  | 15.09$\leq x_{4i4}<$16.59 | 0.53 |
| $S_{5}$ | $T_{1}$ | 4.48$\leq x_{1i5}<$ 5.59 | 2.49 | $T_{2}$ | 9.10$\leq x_{2i5}<$10.10 | 0.19 | $T_{3}$ | 8.15 $\leq x_{3i5}<$ 9.85 | 0.48 | $T_{4}$ | 12.10$\leq x_{4i5}<$13.60 | 0.57 |
|  |  | 5.59$\leq x_{1i5}<$6.78 | 2.47 |  | 10.10$\leq x_{2i5}<$11.10 | 0.15 |  | 9.85 $\leq x_{3i5}<$11.84 | 0.44 |  | 13.60$\leq x_{4i5}<$15.00 | 0.54 |
|  |  | 6.78$\leq x_{1i5}<$7.93 | 2.45 |  | 11.10$\leq x_{2i5}<$12.12 | 0.10 |  | 11.84$\leq x_{3i5}<$13.55 | 0.43 |  | 15.00$\leq x_{4i5}<$16.60 | 0.51 |

Table 3. Inventory information of materials

| Material | Inspection fee | Storage cost | Return price | Penalty | Defect rate | |
| --- | --- | --- | --- | --- | --- | --- |
|  | $d_{t}$*(CNY)* | $K_{t}($*CNY)* | $r_{t}($*CNY)* | $\sigma_{t}$*(CNY)* | $\tilde{\bar{q_{ts}}}$(%) | Parameter |
| $T_{1}$ | 0.009 | 9 | 0.09 | 0.03 | (0.01783,ν1,0.01793) | ν1∼ N(0.01788,0.71) |
| $T_{2}$ | 0.011 | 6 | 0.08 | 0.02 | (0.02105,ν2,0.02118) | ν2∼ N(0.02112,0.88) |
| $T_{3}$ | 0.012 | 3 | 0.03 | 0.03 | (0.00871,ν3,0.0882) | ν3∼ N(0.00876,0.91) |
| $T_{4}$ | 0.006 | 2 | 0.01 | 0.01 | (0.01805,ν4,0.01818) | ν4∼ N(0.01812,0.65) |

Table 4. Distribution cost from supplier to supply hub

|  | Month index ($i$ $i$) | | | | |
| --- | --- | --- | --- | --- | --- |
|  | $i=1$ | $i=2$ | $i=3$ | $i=4$ | $i=5$ |
| $\bar{\gamma_{1i}}$ | $\bar{\gamma_{11}}$~N(21,1.4) | $\bar{\gamma_{12}}$ $\theta_{11}$~N(14,0.8) | $\bar{\gamma_{13}}$ $\theta_{11}$~N(15,0.9) | $\bar{\gamma_{14}}$~N(20,1.3) | $\bar{\gamma_{15}}$~N(15,0.9) |
| $\bar{\gamma_{2i}}$ | $\bar{\gamma_{21}}$ $\theta_{12}$~N(19,1.2) | $\bar{\gamma_{22}}$ $\theta_{12}$~N(13,0.7) | $\bar{\gamma_{23}}$ $\theta_{12}$~N(17,1.1) | $\bar{\gamma_{24}}$ $\theta_{12}$~N(21,1.4) | $\bar{\gamma_{25}}$ $\theta_{12}$~N(17,1.1) |
| $\bar{\gamma_{3i}}$ | $\bar{\gamma_{31}}$ $\theta_{13}$~N(21,1.4) | $\bar{\gamma_{32}}$ $\theta_{13}$~N(14,0.8) | $\bar{\gamma_{33}}$ $\theta_{13}$~N(15,0.9) | $\bar{\gamma_{34}}$ $\theta_{13}$~N(20,1.3) | $\bar{\gamma_{35}}$ $\theta_{13}$~N(15,0.9) |
| $\bar{\gamma_{4i}}$ | $\bar{\gamma_{41}}$ $\theta_{21}$~N(19,1.2) | $\bar{\gamma_{42}}$ $\theta_{21}$~N(23,1.7) | $\bar{\gamma_{43}}$~N(27,2.1) | $\bar{\gamma_{44}}$ $\theta_{21}$~N(31,2.3) | $\bar{\gamma_{45}}$ $\theta_{21}$~N(27,2.1) |

Table 5. Distance among suppliers, supply hubs, and manufacturers (km)

| MFR *n*  Supply hub *t* | MFR 1 | MFR 2 | MFR 3 | MFR 4 | MFR 5 | SUP 1 | SUP 2 | SUP 3 | SUP 4 | SUP 5 |
| --- | --- | --- | --- | --- | --- | --- | --- | --- | --- | --- |
| Supply hub 1 ($D_{1}$) | 189.2 | 180.9 | 124.6 | 169.6 | 304.9 | 244.9 | 171.1 | 257.2 | 146.9 | 106.1 |
| Supply hub 2 ($D_{2}$) | 139.4 | 155.6 | 85.6 | 188.5 | 270.9 | 186.5 | 124.2 | 207.4 | 123.1 | 210.1 |
| Supply hub 3 ($D_{3}$) | 170.5 | 98.8 | 162.7 | 244.2 | 210.3 | 225.4 | 142.8 | 175.3 | 204.5 | 221.7 |
| Supply hub 4 ($D_{4}$) | 126.0 | 169.9 | 88.1 | 232.3 | 206.9 | 111.1 | 43.6 | 133.9 | 173.8 | 266.5 |
